# A solid solution to computational challenges presented by crystal structures exhibiting disorder

**DOI:** 10.1107/S2052252524001684

**Published:** 2024-04-30

**Authors:** Peter R. Spackman

**Affiliations:** a School of Molecular and Life Sciences, Curtin University, GPO Box U1987, Perth, Western Australia 6845, Australia

**Keywords:** disorder, crystal structures, quantum crystallography, structure solution

## Abstract

Crystal structues exhibiting disorder still present a barrier for many computational methods. Dittrich *et al.* [(2024). *IUCrJ*, **11**, 347–358] showcase a unified approach, tackling solid solutions, near symmetry and more.

Crystallography is, in part, an ongoing effort to distil and rationalize interactions and behaviour at the atomic/molecular scale so we may identify their observable impact on macroscopic properties. It is a journey from the invisible world that we can only probe – via poking and prodding with increasingly sophisticated and precise sticks – to the world we perceive around us.

Though it can be very challenging, there is a certain appeal in the endeavour to connect such characteristics of a macroscopic object – a crystal can be held in the hand or examined under a microscope – into a refined, minimalist description characterized by mathematical concepts and constructs like symmetry and infinite lattices. In this manner a real, physical crystal can effectively be encoded into a world of geometric precision and orderly arrangement.

This reduction is not merely a simplification, it is an elegant distillation of natural complexity into comprehensible and manageable terms. It is also more than just a theoretical curiosity; it has implications for the prediction of bulk properties and therefore in praxis. Batteries, pharmaceuticals, an ongoing list of everyday and exotic materials, are all in principle predictable and understandable. The approach is so effective, in fact, that it is routinely applied using surrogate systems to model liquids, amorphous materials and other systems in simulations. The success of computational methods in treating these systems, which are emphatically not periodic, through a periodic lens showcases the fundamental value of reducing a really, really, big, complicated mess to a simple, symmetric and understandable model for analysis and prediction.

Disorder in crystals, then, poses a significant computational challenge; a thorn in the side for those seeking minimalist elegance and a harsh reminder that the reality of the physical world is typically neither elegant nor simple. The challenge is then compounded by further misfortune: crystals often exhibit degrees of disorder, probably even more often than in cases where they do not. Understanding why disorder arises is complex, and the temptation to wave it off with a simple statement like ‘it is entropically favourable’ provides no insight. It can stem from a variety of factors associated with the inherent properties of the materials or the conditions under which the crystals form. In brief, disorder is not just a minor inconvenience; it poses substantial challenges in accurately modelling and predicting the properties of bulk materials, and it is necessary to understand disorder before we can understand the real world. Indeed, many macroscopic properties directly result from the disorder (Welberry & Goossens, 2014 ▸), and would not occur in perfect crystals.

Thankfully, even within the realm of disordered structures, there are degrees of order. Sometimes it is as simple as a 50–50 mix of two orientations of a functional group; at other times there is effectively an infinite number of orientations of a solvent or trapped gas molecule, and sometimes we have solid solutions where molecules are effectively interchangeable at specific crystallographic sites. There is also a link to higher *Z*′ crystal structures, which have been demonstrated to frequently be symmetry ‘near misses’ with regard to lower *Z*′ *i.e.* higher symmetry structures (Brock, 2016 ▸).

Crystallographers, practical as they are, have been tackling disorder for a long time. However, for many computational folks (including myself) if the material is disordered, we would really prefer not to deal with the hassle. It would be unfair to imply that no work has been done at all to resolve the challenges. Indeed, the problem may be circumvented by treating a system as ordered, evaluating local defects in infinite systems or a variety of other approaches. But I believe the most common choice is likely to be simple: just avoid working on systems that exhibit disorder!

It is in this context that providing the community with clear methods to rationalize the order within disordered structures is invaluable for many real-world scientific problems, and that is exactly where Dittrich *et al.* (2024 ▸) provide an excellent step in the right direction in the latest issue of **IUCrJ** .

Their concept of ‘archetype crystal structures’ will no doubt help in wrangling disorder in many computational methods and applications. Further, the authors provide practical advice and concrete examples to help resolve many thermodynamic problems associated with several varieties of disordered crystals, including solid solutions. This is accomplished in a unified manner with analysis of polymorphism in crystals – an area itself once considered niche but increasingly seen as normal. As it turns out, we found different polymorphs almost everywhere once we started really looking for them (Bernstein, 2020 ▸).

The above work in particular showcases tangible applications of the concept of archetype structures to high-*Z*′ structures, and molecules in special positions which, like disordered systems, many scientists would prefer to avoid. In this manner it serves both as an excellent exposition and showcase of the practical value of the concept.

That is not to say the work is done – there are still various factors associated with free energies which themselves have an impact on the explanation of these phenomena (including entropy, vibrational energies *etc*.) but the work here should find a variety of uses and prove valuable both within the crystallographic community and to the broader chemical, physics and materials communities.

In my view, the particular value of works such as this lies in the simple fact that the use of the concepts presented should reduce the difficulty of analysing many real-world problems. It is wonderful when we, as scientists, can find lower-hanging fruit; it is perhaps even better when others provide us with a ladder to reach up to new heights.
